# Supplementary material for: Analysis of PPARγ Signaling Activity in Psoriasis
Source: Int J Mol Sci. 2021 Aug 10;22(16):8603. doi: 10.3390/ijms22168603 (PMC8395241; doi:10.3390/ijms22168603)
Supplement: Supplementary file 1 [file ijms-22-08603-s001.zip › Supplemental materials_Analysis of PPARg signaling activity in psoriasis/Pathway models/Models images and html files/PPARG inhibits phenotypes assotiated with psoriasis/36167.html]

neoplasm


# Disease neoplasm

|  |  |
| --- | --- |
| URN | urn:agi-meshdis:Neoplasms |
| Total Entities | 33 |
| Connectivity | 41397 |
| Name | neoplasm |

---

|  |  |
| --- | --- |
| ChildConcepts | cancer |
|  | mixed neoplasm |
|  | preneoplastic condition |
|  | embryonal and mixed neoplasms |
|  | post-traumatic neoplasm |
|  | radiation-induced neoplasm |
|  | benign neoplasm |
|  | metastasis |
|  | precancer and cancer-in-situ |
|  | incidentaloma |
|  | neoplasms subdivided by anatomical site |
|  | congenital neoplasm |
|  | fetal neoplasm |
|  | malignant transformation |
|  | neoplasm of uncertain behavior |
|  | paraneoplastic syndrome |
|  | tumor microenvironment |
|  | tumor necrosis |
|  | tumor spheroid |
|  | neoplastic process |
|  | tumor hypoxia |
|  | germ cell and embryonal neoplasms |
|  | bilateral neoplasm |
|  | tumor syndrome |
|  | multiple primary neoplasm |
|  | gingival carcinoma |
|  | cystic, mucinous, and serous neoplasms |
|  | maxillary neoplasm |
|  | local neoplasm recurrence |
|  | neoplasms by histologic type |
|  | hormone-dependent neoplasm |
|  | rare neoplastic disease |
|  | tumor escape |

---

|  |  |
| --- | --- |
| Pathway | Androgen Receptor/Akt Signaling |
|  | Proteins with Altered Expression in Cancer-Associated Sustaining of Proliferative Signaling |
|  | Genes with Mutations in Cancer-Associated Sustaining of Proliferative Signaling |
|  | Genes with Mutations in Cancer-Associated Evading of Growth Suppressors |
|  | Proteins with Altered Expression in Cancer-Associated Evading of Growth Suppressors |
|  | Proteins with Altered Expression in Cancer-Associated Resisting to Cell Death |
|  | Genes with Mutations in Cancer-Associated Resisting to Cell Death |
|  | Genes with Mutations in Cancer-Associated Enabling of Replicative Immortality |
|  | Proteins with Altered Expression in Cancer-Associated Enabling of Replicative Immortality |
|  | Genes with Mutations in Cancer Metabolic Reprogramming (Respiratory Chain) |
|  | Tumor Infiltrating Macrophages in Cancer Progression and Immune Escape |
|  | Proteins with Altered Expression in Cancer Immune Escape |
|  | Genes with Mutations in Cancer Immune Escape |
|  | Genes with Mutation in Cancer-Associated Dysregulated DNA Repair |
|  | Proteins with Altered Expression in Cancer-Associated Dysregulated DNA Repair |
|  | Genes with Mutations in Cancer-Associated DNA Methylation |
|  | Genes with Mutations in Cancer-Associated Histone Deacetylation |
|  | Proteins with Altered Expression in Cancer-Associated Histone Methylation |
|  | Genes with Mutations in Cancer-Associated Histone Methylation |
|  | Proteins with Altered Expression in Cancer-Associated Histone Deacetylation |
|  | Proteins with Altered Expression in Cancer-Associated DNA Methylation |
|  | PPARG inhibits phenotypes assotiated with psoriasis |
|  | Neighbors of neoplasm |

---

|  |  |
| --- | --- |
| MedScan ID | 9016100 |

---

|  |  |
| --- | --- |
| Alias | Neoplastic Growth |
|  | neo-plastic |
|  | Neoplasm |
|  | neoplasias |
|  | Neoplasms of Unspecified Nature |
|  | Neoplastic Growths |
|  | Neoplastic Disease |
|  | Neoplastic Diseases |
|  | Neoplasms |
|  | neoplastic |
|  | Neoplasia |
|  | Tumors |
|  | Neoplasm of Unspecified Nature |
|  | Tumor |
|  | cytomatosis |
|  | acral tumor |

---

|  |  |
| --- | --- |
| MeSH ID | D009369 |
|  | M0014585 |

---

|  |  |
| --- | --- |
| MeSH Heading | Neoplasms |

---
